# Transcriptional and physiological analyses of reduced density in apple provide insight into the regulation involved in photosynthesis

**DOI:** 10.1371/journal.pone.0239737

**Published:** 2020-10-12

**Authors:** Junqiang Niu, Ming Ma, Xiaoning Yin, Xinglu Liu, Tie Dong, Wentai Sun, Fuxia Yang

**Affiliations:** Institute of Fruit and Floriculture Research, Gansu Academy of Agricultural Sciences, Lanzhou, Gansu Province, People’s Republic of China; Institute for Horticultural Plants, China Agricultural University, CHINA

## Abstract

Different densities have a great influence on the physiological process and growth of orchard plants. Exploring the molecular basis and revealing key candidate genes for different densities management of orchard has great significance for production capacity improvement. In this study, transcriptome sequencing of apple trees was carried out at three different sampling heights to determine gene expression patterns under high density(HD) and low density(LD) and the physiological indices were measured to determine the effect of density change on plants. As a result, physiological indexes showed that the content of Chlorophyll, ACC, RUBP and PEP in the LD was apparently higher than that in control group(high density, HD). While the content of PPO and AO in the LD was noticeably lower than that in the HD. There were 3808 differentially expressed genes (DEGs) were detected between HD and LD, of which 1935, 2390 and 1108 DEGs were found in the three comparisons(middle-upper, lower-outer and lower-inner), respectively. 274 common differentially expressed genes (co-DEGs) were contained in all three comparisons. Functional enrichment and KEGG pathway analysis found these genes were involved in Carbon fixation in photosynthetic organisms, Circadian rhythm, Photosynthesis − antenna proteins, Photosynthesis, chlorophyll metabolism, Porphyrin, sugar metabolism and so on. Among these genes, *LHCB* family participated in photosynthesis as parts of photosystem II. In addition, *SPA1*, *rbcL*, *SNRK2*, *MYC2*, *BSK*, *SAUR* and *PP2C* are involved in Circadian rhythm, the expression of genes related to glycometabolism and hormone signaling pathway is also changed. The results revealed that the decrease of plant density changed the photosynthetic efficiency of leaves and the expression of photosynthesis-related genes, which provide a theoretical basis for the actual production regulation of apples.

## Introduction

A large number of mature arborized apple orchards have appeared many problems, such as crown canopy closure, tree shape disorder, serious diseases, low yield and poor quality, which seriously restrict the healthy and sustainable development of apple industry [1, 2]. Scientific adjustment of orchard population structure is an important link in apple production with high quality and efficiency, especially for arborized closed orchards. The adjustment can significantly improve light distribution, fruit yield and quality [3, 4].

Different densities have different effects on plants growth, physiology and fertilizer utilization. Studies on maize found that high-density plants reduced the ability of plants to use nitrogen from the soil [5]. The increase of spacing interval of trees will reduce disease and the use of pesticides, which become a potential integrated pest management method. The tree spacing with a certain radius had a notable passive impact on the incidence of FPR on individual trees [3]. Studies found that different spacing have a great influence on Cauliflower production [6]. Reduce the current-year and one-year-old conifers density can decline δ18O, increase δ13C and the net photosynthetic rate, the dry matter distribution was greatly affected by the row spacing and trees planted at low density had lower mortality than those planted at high density before the crown closed [7, 8]. In addition, forests with moderate decrease density are more conducive to carbon sequestration than those without thinning accelerating and large-scale reduced forest density can improve resilience of forest, water and maintain ecological balance [9–11].

With the development of "next generation" sequencing technology, transcriptome has been widely used to study the influence of environment on plant growth and development [12–14]. Cassava intercropping with other crops caused varying degrees of shade, leading to a decline in yield. The comparative transcriptome analysis of cassava leaves under full day and natural shade conditions found that the gene expression profiles in both cases showed similar developmental changes, the expression of hormone related transcription factors and genes were influenced by shade [15]. The transcriptome analysis of poplar and maize with different densities revealed the differential expression of genes related to plant hormones, photosynthetic complexes and photosynthetic antenna proteins [16, 17]. However, there are few studies on the effect of planting density on gene expression, which need more research.

*Malus Domestica* “Changfu 2 Fuji” as the test material, the method of inter-strain thinning was adopted with 50% reduced plant density, combined with lifting, falling head, opening angle. The physiological indexes of chlorophyll, ascorbic acid, ascorbic acid oxidase, polyphenol oxidase and ribose-1,5-diphosphate of before and after density reduced were analyzed, meanwhile the differentially expressed genes were analyzed by transcriptome. Therefore, the aim of this study is to identify density-related genes and explore the molecular mechanism of low density on apple growth and development.

## Materials and methods

### Plant material and growth conditions

In mid-January 2017, it was carried out in Wumiao Village Experimental Park, Chengchuan Town, Jingning County, Gansu Province, China. The orchard covers an area of 7.8 mu, the tree age is 15 years, planting density is 3×4 m and the variety is Changfu No. 2 Fuji apple. The shape of the tree is improved spindle, with a trunk height of 50–60 cm, tree height of 3.8–4.1m, a few main branches of 12–14, and the main branch angle is about 75°. The orchard ground is covered with black plastic film, the fruit is bagged, spraying 6 times a year, and the orchard management level is high. 40 trees were evenly divided into two groups (the high density group as the control and the low density group as density decreased group) along the east-west trend, the low density group was treated by thinning with separate trees, and the pruning amount accounted for about 30% of the total amount of branches combined with lifting drying (100–120 cm), falling head (300–320 cm), opening angle (90 degrees of main branch angle), and septal thinning with 50% reduced plant density. The other group was used as control, Septal thinning, drying, dropping and angle-opening measures were not adopted, and only pruning was carried out on the tree body with the amount of about 30%.

### Treatments and experimental design

In mid-August of 2018, 5 trees with moderate growth potential and basically the same growth potential were respectively selected from 2 groups as test materials. The tree body is divided into two layers from the bottom to the top along the trunk direction, namely the lower layer (less than 1.5 m from the ground) and the upper and middle layer (more than 1.5 m from the ground). Since the tested tree is an improved spindle shape, wide at the bottom and narrow at the top, the lower layer is divided into two parts along the crown diameter direction: the inner part (< 1m from the central leading trunk) and the outer part (> 1.0m from the central leading trunk). The upper-middle layer no longer divided along the direction of the crown. This divides the test tree into three parts, namely: middle-upper (A1), lower-outer (A2), and lower-inner (A3).50 healthy functional leaves without pests and diseases, mechanical damage and robustness were uniformly taken in different directions and parts, Storage by liquid nitrogen.

### Measurement of plant physiological parameters

The determination of Chlorophyll, ACC, AO, RUBP, PEP and PPO was performed by ELISA kit. Adding test antibodies labeled samples, standard substances, and HRP to the Micropores pre-coated with capture antibodies, and then incubated and thoroughly washed. The substrate TMB was converted to blue under the catalysis of peroxidase and finally turned yellow under the action of acid. The depth of color has a positive correlativity with the concentration of the substance in the sample. The absorbance (OD) of the samples was measured at 450 nm by Microplate reader, and then the concentration of the sample was calculated [18].

### RNA extraction and RNA-seq

Total RNA were extracted from leaves with Trizol reagent (Invitrogen, USA) following the method described by Yan-Xiu W [19]. The total RNA extraction was divided into two parts: one for RNA sequencing and the other for real-time PCR. The RNA sequencing was done at Beijing Ori-gene Technology Co. Ltd., Beijing, China, using the illumine HiSeq 4000 platform (Illumina, San Diego, CA, USA). Each sample produced more than 6 GB raw data. Remove the low-quality reads with unknown nucleotides or low matching degree from the original sequencing data to get clean data [20].

### Analysis of differentially expressed genes (DEGs)

Differential gene expression analysis was conducted for different samples based on the DESeq R package. The software package allows statistical analysis using a negative binomial distribution model [21]. In order to check the false detection rate, the p value was adjusted according to the Benjamini and Hochberg methods, where a p < 0.05 is accepted to represent differentially expressed genes(DEGs) [22]. The GO(gene ontology) enrichment analysis of the DEGs was conducted according to the Metascope (http://metascape.org/gp/index.html), and GO terms with q < 0.05 were regarded as significantly enriched [23, 24].

### Quantitative RT-PCR analysis (qRT-PCR)

The total RNA was extracted using Trizol reagent (Invitrogen, USA) according to the manufacturer’s protocol. The first-strand cDNA was synthesized using the Revert Aid First Strand cDNA Synthesis Kit (Thermo, USA) and then used as a template for qRT-PCR reactions. The qRT-PCR was performed by ABI 7300 (ABI, USA). The 18S was used as an internal standard. All primers for qRT-PCR are listed in S1 Table. Reactions were performed by the FastStart Universal SYBR Green Master(Rox) (Roche, Switzerland) following the manufacturer’s protocol. The PCR parameters were set as follows: 95°C10 min, 40 cycles of 15 s at 95°C, and15 s at 60°C. Each sample was repeated three times.

### Statistical analysis

The analysis of variance(ANOVA) of collected data were analyzed by the aid of SPSS(Statistical Product and Service Solutions, Version 22, IBM, Armonk, NY, USA). The treatment means were performed by Duncan’s multiple range test (p < 0.01) and the results were represented by the mean ±standard error of mean (SEM)of three repetitions.

## Results

### Effect of light durations on plant physiological indexes

**Fig 1:**
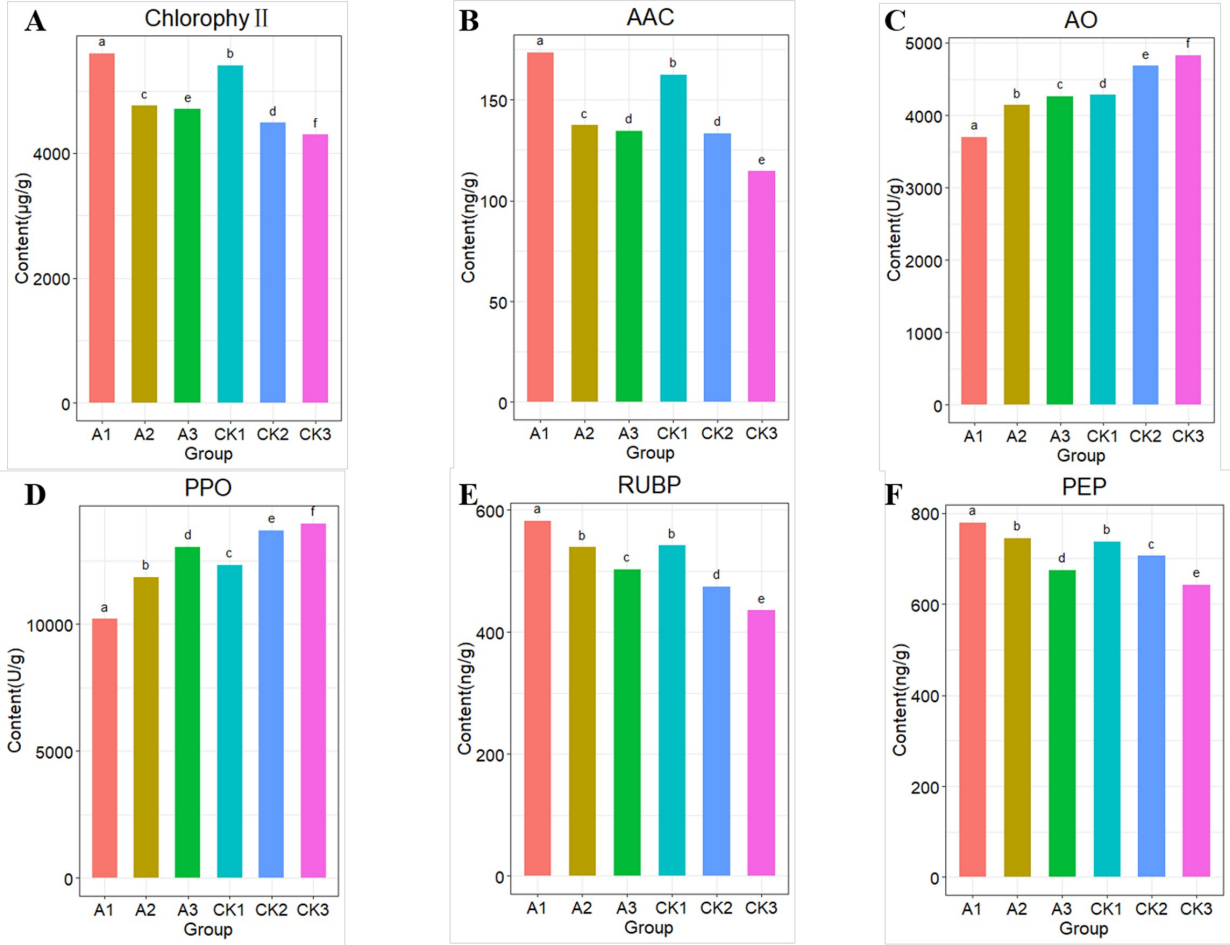
Photosynthetic related physiological indexes of apple leaves after reduce density. Lower case letters above the column in the figure indicate significant differences between treatments at the P< 0.05 level.

The physiological indicators of various parts of leaves before and after thinning were detected as follows (Fig 1): Chlorophyll, ACC, RUBP and PEP in leaves of thinning group were higher than those of control group. On the contrary, the content of PPO and AO in thinning group was lower than that in the control group, the difference was significant (P<0.05). The results of comparison among the three treatment groups showed that: the contents of Chlorophyll, ACC, RUBP, and PEP were A1 > A2 > A3, while the contents of PPO and AO were opposite, A1 < A2 <A3, and the differences were significant. In addition, the other 9 physiological indexes were supplemented in S2 Table.

### Overview of RNA sequencing

Among the 12 sequencing samples (12 samples were divided into 2 groups, each group contained six samples), an average of 47.2 million total sequencing reads for each sample were obtained (S3 Table). HISAT software filtered sequences were selected to perform genomic positioning analysis. An average of 43.6 million reads was matched to the genome in each group. Each library produced clean reads was aligned to the recently released Malus Domestica (apple) reference genome(GCA_002114115.1) [25]. An average of 92.4% of clean reads was aligned to the reference genome, and there is no multiple comparison in the comparative position of reads.

### Results of the screen for the DEGs between treatments

**Fig 2:**
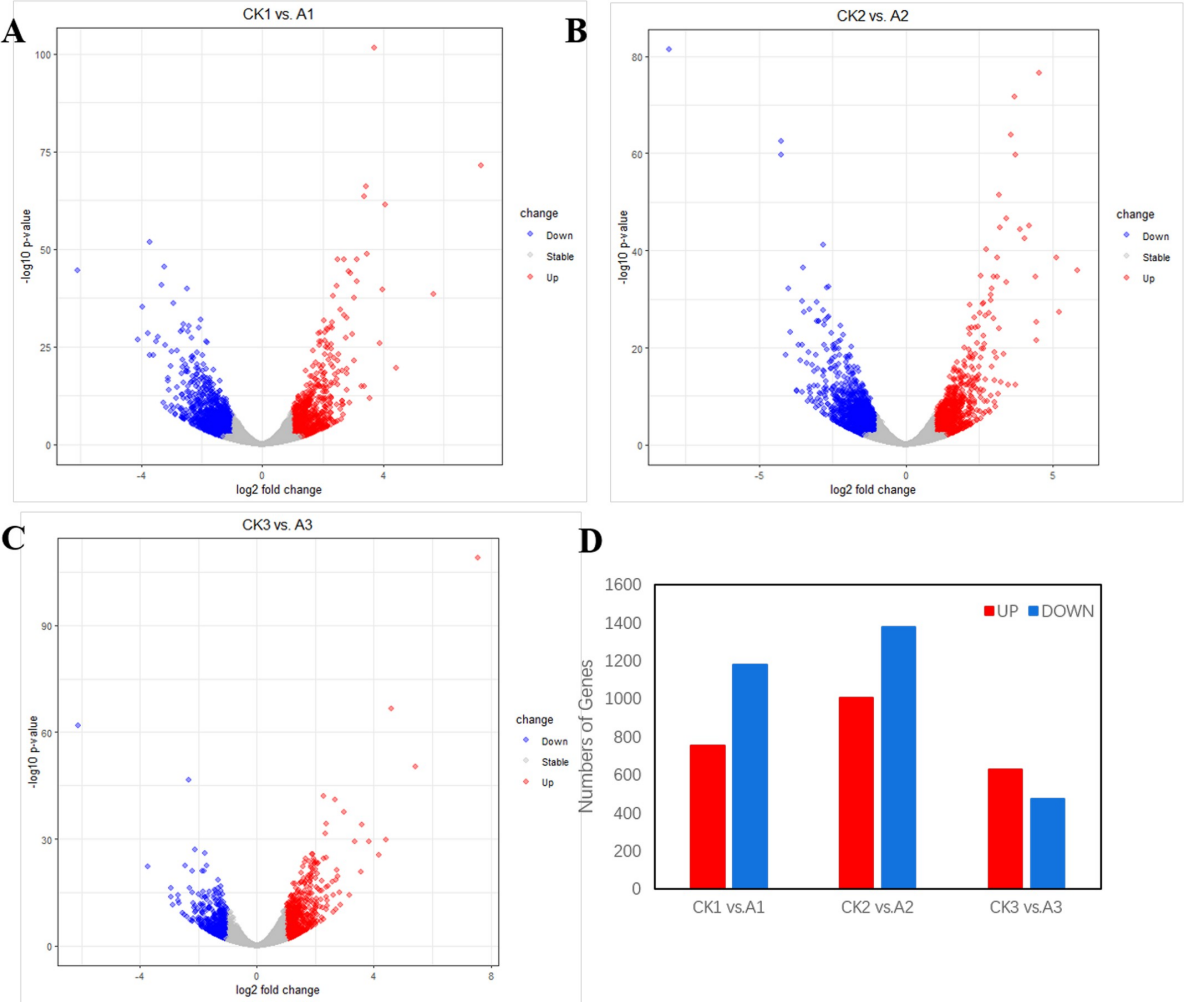
Expression profiles of mRNAs in A1, A2 and A3 compared with control groups. The Vocalo plot of (A), (B), (C) represent the expression of all genes in A1 vs CK1, A2 vs CK2, and A3 vs CK3. Different expressed genes were marked with red and blue, Red indicates upregulated genes, blue is down regulated. (D) Histogram of DEGs in A1 vs CK1, A2 vs CK2, and A3 vs CK3.

Based on the assembly results of the transcript, the sequence and annotation information of the gene were determined, and new genes were screened for statistics and annotation. A total of 55,169 genes were counted, of which 52,741 were aligned to the genome and were known genes. The remaining 2428 was Novel Gene. The 52,741 genome matched genes were normalized using the reads per kilobases per million reads (FPKMs) method [26]. FPKM were obtained to analyze the differences in gene expression of each group, and then the FPKM of each treatment group was compared with the control (CK) plants (S4 Table). The criteria for screening DEGs is log2| (fold change) | > 1 and q-value <0.005. The results of gene expression focused on were as follows: 756 genes were down-regulated and 1179 genes were up-regulated in the first comparison A1 vs. CK1;1009 genes were down-regulated and 1381 genes were up-regulated in the second comparison A2 VS CK2, while 631 genes were down-regulated and 477 genes were up-regulated in the third comparison A3 VS CK3 (Fig 2).

### Venn and cluster analysis of DEGs among the treatments

**Fig 3:**
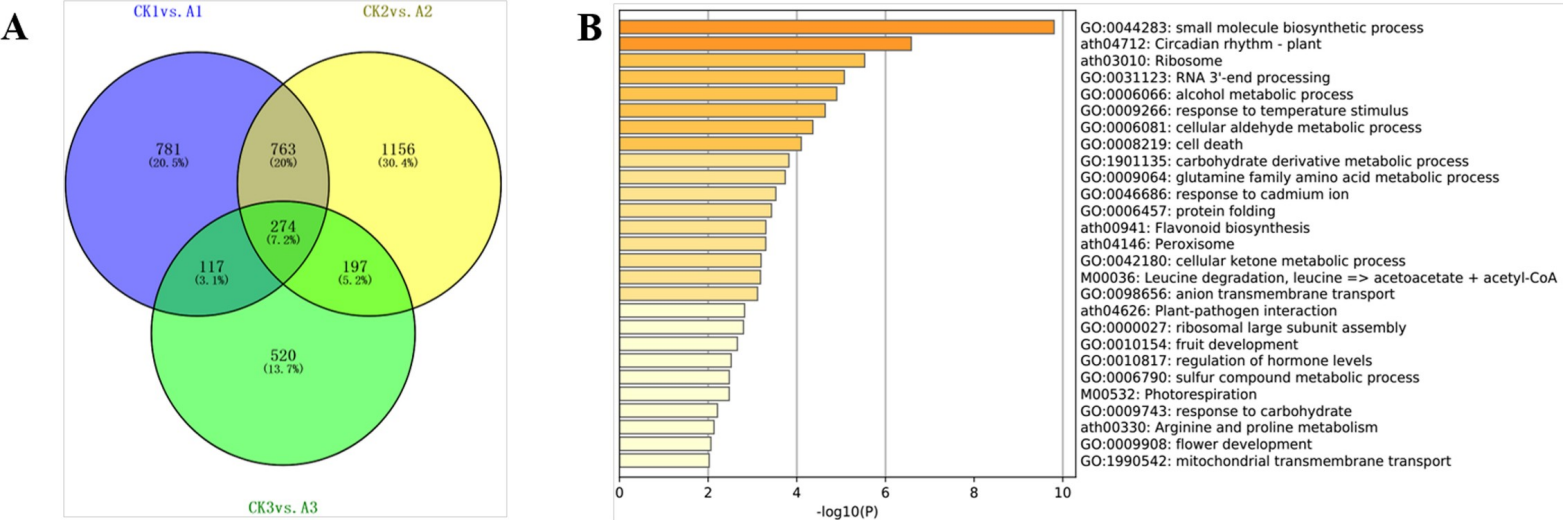
Venn diagram and cluster analyses of DEGs in CK1 vs A1, CK2 vs A2, and CK3 vsA3. (A) Venn diagram showing the overlapped DEGs in three comparison groups; (B) Bargraph of enriched top GO-term and KEGG pathways of co-DEGs, colored by p-values.

781 differentially expressed genes specifically existed in the first comparison CK1 vs. A1, 1156 DEGs in the second comparison CK2 vs. A2 and 520 DEGs in CK3 vs. A3 comparisons, respectively; 763 DEGs existed both in CK1 vs. A1 and CK2 vs. A2 comparison, 197 DEGs in comparison CK2 vs. A2 and CK3 vs. A3, and 117 in CK3 vs. A3 and CK1 vs. A1 comparison, respectively; and 274 common differentially expressed genes (co-DEGs) were contained in all three comparisons (Fig 3A).

### Functional enrichment and KEGG pathway analysis of co-DEGs

Gene Ontology (GO) and Kyoto Encyclopedia of Genes and Genomes (KEGG) pathway enrichment analysis of DEGs in plants were performed to investigate the distribution of cellular components, molecular functions and biological processes in order to elucidate the manifestations of DEGs. Correspondingly, 756 down-regulated DEGs in the first comparison A1 VS CK1 were enriched in 247 Go-items and 1179 up-regulated genes in 253 Go-items; in the second comparison A2 VS CK2, 1009 down-regulated DEGs were enriched in 279 and 1381 up-regulated genes in 286 Go items; while in the third comparison A3 VS CK3, 631 down-regulated genes were enriched in 96 Go items and 477 up-regulated DEGs in 284 Go-items.

**Fig 4:**
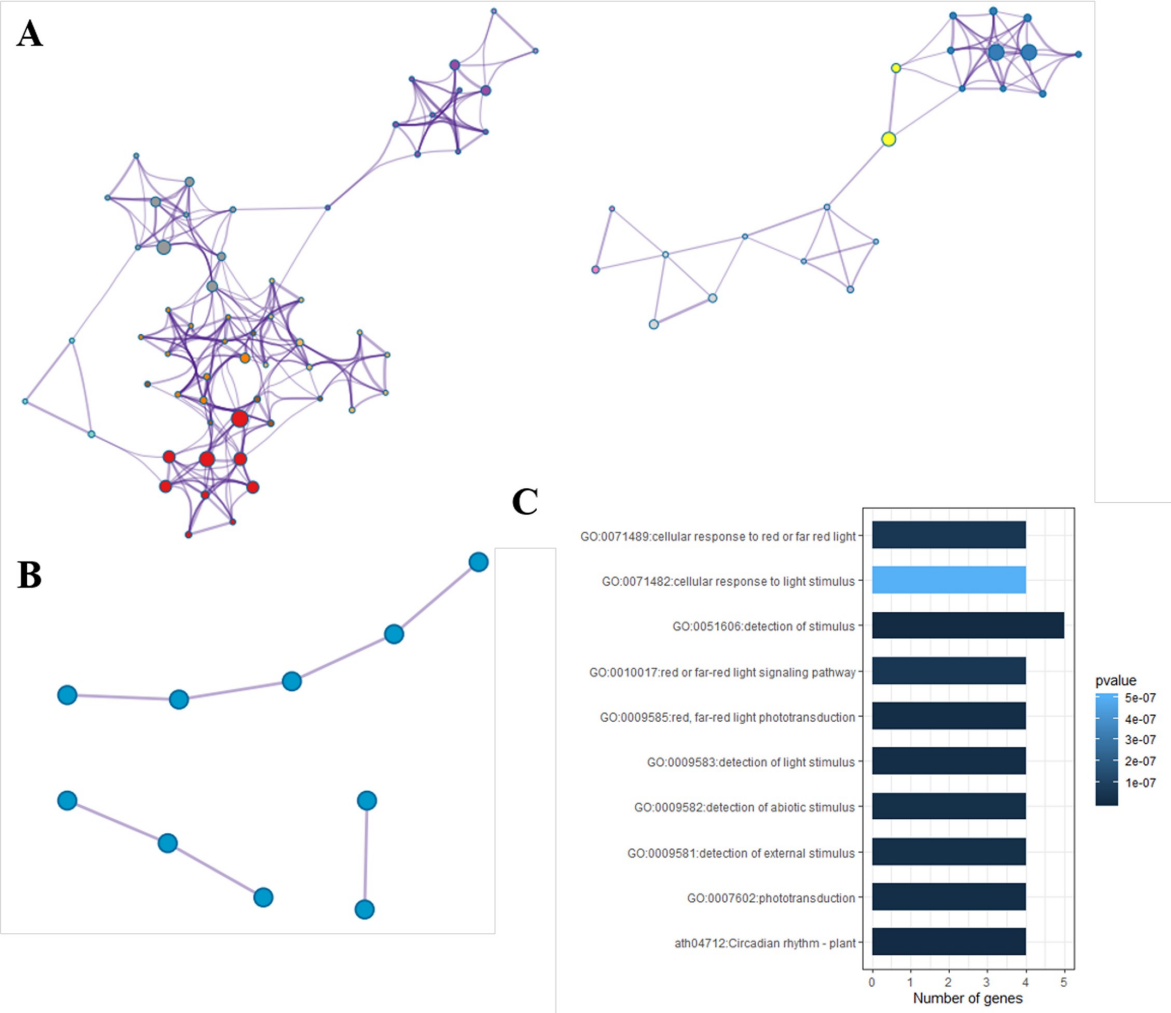
Metascape analysis of co-DEGs. (A) Network of enriched terms: colored by cluster ID, Threshold: 0.3 kappa score; similarity score > 0.3. (B) Module analysis: the three best-scoring terms by p-value. (C) Pathway and process enrichment in the three best-scoring terms. Heatmap colored by P-values.

274 common differentially expressed genes in three comparisons were enriched in 147 GO items and 18 KEGG pathway. To further capture the relationships between the GO and KEGG terms, Metascape analysis showed the top 27 clusters of enriched sets (Fig 3B). KEGG analysis showed that DEGs in A1 VS CK1, A2 VS CK2 and A3 VS CK3 groups were significantly enriched in 18, 22 and 20 metabolic pathways, respectively. These pathways are concentrated in Fatty acid elongation, Circadian rhythm, Photosynthesis, Photosynthesis-antenna proteins, Porphyrin and chlorophyll metabolism, Plant hormone signal transduction, Pentose phosphate pathway, Nitrogen metabolism, its important physiological process related to photosynthesis and substance metabolism. In addition, 66 differentially expressed genes were significantly enriched in plant hormone signal transduction (S5 Table). The genes associated with auxin pathway are *AUX*, *IAA*, *SAUR*, *ARF*. Gene *ARR-B* related to cytokinine, abscisic acid related-*PP2C*, *SNRK2*, *ABF*, *PYL*, *ETR*, *ERF2*. Gene *ETR*, *ERF2*, *EBF1_2* associated with ethylene (S1 Fig). Jasmonic acid related *JAZ*, *MYC2*, and Brassinosteroid related *BSK* genes, these 17 genes were differentially expressed in CK1 vs. A1, CK2 vs. A2, and CK3 vs. A3. *MD12G1156600* (*BSK*), *MD09G1176100* (*SAUR*) and *MD15G1195800* (*PP2C*) were differentially expressed in LD and HD. Interestingly, they were enriched in a transcription factor MYC2. For each co-DEGs protein-protein interaction enrichment analysis has been carried out with the following databases: String. The resultant network contains the subset of proteins that form physical interactions with at least one other member in the list (Fig 4A). MCODE made model analysis of go and pathway on enrichment, and found 10 significantly enriched (P < 0.05) go term or pathway, top 10 was shown in the Fig 4B. Among these enriched GO terms, there are circle rhythm–plant, red, far red light photo translation, detection of light stimulus and so on, which are all related to photosynthesis. Top three best-scoring terms by p-value have been retained as the functional description of the corresponding components as showed in Fig 4C.

### qRT-PCR validation of RNA-seq results

**Fig 5:**
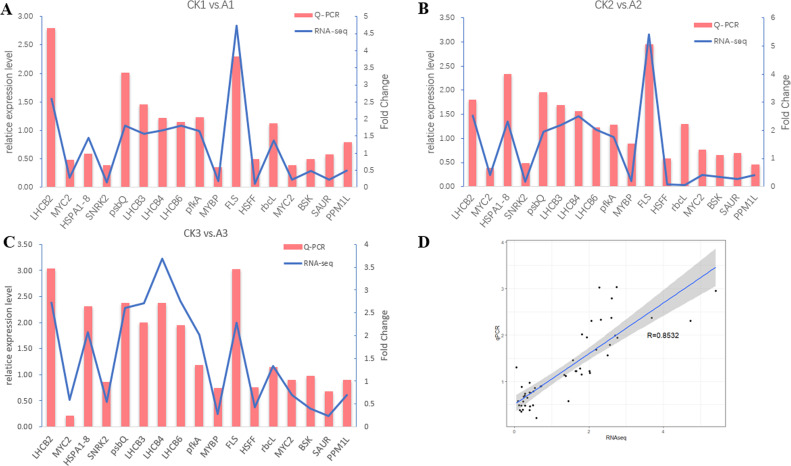
qRT-PCR analysis of differentially expressed genes. (A), (B), (C) represent the expression level of transcriptome sequencing and QRT PCR of 17 randomly selected genes in A1 vs. CK1, A2 vs. CK2 and A3 vs. CK3. The relative gene expression levels analyzed by QPCR were showed as Red histogram. The corresponding expression data of RNA-seq were showed as blue line. The x-axis represents the 17 randomly selected genes. Bars represent SE (n = 3). (D) Comparison between the log2 of the gene expression ratios obtained from RNA-seq data and qRT-PCR results.

In order to verify the reliability of RNA sequencing results, 17 significantly differential expressed genes were randomly selected for qPCR. The results showed that the differentially expressed genes in the sequencing data were consistent with the expression trend in the qPCR (Fig 5). In addition, there was a strong correlation between the results of qRT-PCR and the data generated by RNA-seq, Pearson correlation coefficient RD = 0.8532 (Fig 5D).

## Discussion

Orchard group structure adjustment is an important part of apple high-quality and efficient production, especially for arbor closed orchards. Numerous studies have shown that reducing the density affects the physiological processes of plants through photosynthesis, fertilizer metabolism, material metabolism, and diseases. However, the molecular basis of the effect of reducing the density on apple growth and related genes is unclear. In this study, the dynamic changes of transcripts were associated with thinning, several candidate genes related to reducing the density affecting apple growth physiology were found, and proposed that thinning was a potentially important pathway.

Ascorbic acid oxidase (AAO) is a kind of plant blue-copper protein, whose activity was found to be proportional to light intensity [27]. The enzyme PPO known as substrates in the vacuole, is targeted to the thylakoid lumen and plays a role in photosynthesis [28, 29]. The results of physiological indices of six groups indicated that compared with the same position of control group trees, the contents of enzymes related to photosynthesis and glucose metabolism were changed in the thinning group (A1, A2, A3). This suggests that thinning does affect the physiological processes of photosynthesis and metabolism of plants. Interestingly, this is consistent with many previous studies [30].

There were 6 co-DEGs in the three comparisons were enriched in the pathway Photosynthesis—antenna proteins, all enriched DEGs belong to the LHCB family, the expression of *LHCB 2*, *LHCB 3*, *LHCB 4* and *LHCB 6* was up-regulated in this study. Many studies have found that the LHCB family is a component of light and system, which is closely related to photosynthesis [31–35]. Furthermore, 5 co-DEGs in the three comparisons were enriched in Porphyrin and chlorophyll metabolism pathway, the oxygen-evolving complex of eukaryotic photosystem II (PSII) consists of PsbO, PsbP, PsbQ and PsbR four extrinsic subunits. Arabidopsis results provided evidence that PsbR is an important link for stable assembly of the oxygen-evolving complex protein PsbP, whereas the effects on the assembly of PsbQ are probably indirect [36]. Interestingly, the expression of PsbQ was up-regulated in this study. Furthermore, module analysis found that 9 GO term and 1 KEGG pathway were enriched in the top three modules, and the genes enriched in were Elf3, TOC1, SnRK1, COP1, pif4 and Spa1. the PIFs play a central role in repressing photomorphogenesis. In addition studies in Arabidopsis found that the interaction of ELF3 and (Phytochrome-Interacting Factors, PIFs) PIF4 proteins prevented PIF4 from activating its transcriptional targets. PIF4 overexpression leads to instability of Elf3 protein, which is indirectly mediated by negative feedback regulation of Phytochrome B (phyB). phyB is needed for ELF3 accumulation in the light. Interestingly, this research result is consistent with our research, in the top3 model, Elf3 down regulated while PIF4 up-regulated [37]. Moreover, TOC1 and *SPA1* plays an important role in integrating light signals to control circadian rhythm and morphogenesis [38, 39]. Cryptochrome is a blue light receptor that has been shown to be involved in the light regulation of gene expression. An Arabidopsis study shows that *SPA1* interacts with the downstream cryptochrome circadian rhythm regulator 2 (*CRY2*), which regulates blue light to suppress flowering regulator COP1-dependent proteolysis [40].

In addition, the expression of some genes related to glycometabolism and hormone signaling pathway have changed in this study. *RBCL* expression was up-regulated and the *SNRK2* was down-regulated in group A2 vs CK2. Sucrose nonfermenting 1 (SNF1)-related protein kinase 2s (SnRK2s) is key parts of abscisic acid (ABA) signaling pathways [41]. Previous studies found that *MYC2* was related to ABA signaling and JA signaling pathway [42–47]. There is a direct interaction between the ABA receptor PYL6 (RCAR9) and the transcription factor MYC2 in the gene and mechanism of ABA-JA crosstalk [48]. Small auxin-up RNAs (SAURs) are the early auxin-responsive genes in plants and provided a link between plasma membrane H-ATPase (PM H-ATPase) and auxin [49, 50]. The plant hormone abscisic acid (ABA) is a developmental signal that responds to environmental stimuli.2C type protein phosphatases (*PP2Cs*) is closely related to abscisic acid (ABA) signaling pathway and play a role through the negative regulation of ABA response [51, 52]. *SUAR*, *IAA* and *PP2C* involved in plant hormone signal transduction pathway were higher in HD than LD, which was consistent with the findings in poplar [16]. In addition, the Aux / IAA related genes were down regulated under HD when compared to LD. Our transcriptome sequencing experiments showed that *BSK*, *SAUR* and *PP2C* were differentially expressed in all of the three comparisons, and *MYC2* was differentially expressed in the second comparison (CK2 vs. A2) and the third comparison (CK3 vs. A3).

## Conclusions

The decrease of density significantly increased the contents of Chlorophyll, ACC, RuBP and PEP in apple leaves. Chlorophyll and ACC were involved in photosynthesis, RuBP and PEP were involved in Glucose metabolism. The physiological process of photosynthesis and metabolism is affected by the decrease of plant density. Meanwhile, transcriptome analysis showed that *Lhcb2*, *Lhcb3*, *Lhcb4*, *Lhcb6*, *chld*, *PSBR*, *psbq*, *Spa1*, *rbcL*, *SnRK2*, *myc2*, *Saur* and *PP2C* were expressed differently in treatment plant compared with control group. Key genes and pathways related to Photosynthesis, Circadian rhythm, Carbon fixation in photosynthetic organisms, Photosynthesis-antenna proteins, Porphyrin and chlorophyll metabolism, respectively. This suggested that decrease in plant density alters the expression of photosynthesis-related genes, and affects the growth of plants. Moreover, the validation of 17 randomly selected co-DEGs by qPCR implied that the results of transcription sequencing were reliable. The study has furthered our understanding of the molecular mechanism of the effect of density related genes on apple growth and development, and provided valuable reference of population structure adjustment on quality and production capacity of apple in closed orchard.

## Supporting information

S1 Table Primers used for qRT-PCR analysis of genes selected from the RNA sequencing data. (XLSX)

S2 Table Photosynthetic related physiological indexes of apple leaves after reduce density. (XLSX)

S3 Table Summary of the RNA sequencing data. (XLSX)

S4 Table Statistical distribution of FPKM interval values in each sample. (XLSX)

S5 Table KEGG analysis of DEGs in LD and HD. (XLSX)

S1 Fig Plant hormone signal transduction pathway and related genes. A. Differentially expressed genes enriched in plant hormone signal transduction. Red represents up-regulated genes maped to this node, green represents down regulated genes maped to this node, yellow represents both up-regulated genes and down-regulated genes maped to this node. B. Cluster heat map of differentially expressed genes related to plant hormone signal transduction pathway. The horizontal axis showed the name of different comparison groups, and the vertical axis represent different genes.
(TIF)
