# Correction: Transcriptional and physiological analyses of reduced density in apple provide insight into the regulation involved in photosynthesis

**DOI:** 10.1371/journal.pone.0244012

**Published:** 2020-12-09

**Authors:** Junqiang Niu, Ming Ma, Xiaoning Yin, Xinglu Liu, Tie Dong, Wentai Sun, Fuxia Yang

There is an error in the Funding statement. The correct Funding statement is as follows: This research was supported by the National Natural Science Foundation of China (31760556), Agricultural science and technology innovation project of Gansu Academy of Agricultural Sciences (2016GAAS14); National key Research and Development plan project (2016YFD001135);Science and technology research project of apple industry in Gansu Province (GPCK2011-1); Fruit tree science observation station project in Northwest China of Ministry of agriculture (10218020).The funders had no role in study design, data collection and analysis, decision to publish, or preparation of the manuscript.
