# Supplementary figures and images for: Transcriptional and physiological analyses of reduced density in apple provide insight into the regulation involved in photosynthesis
Source: PLoS One. 2020 Oct 12;15(10):e0239737. doi: 10.1371/journal.pone.0239737 (PMC7549834; doi:10.1371/journal.pone.0239737)

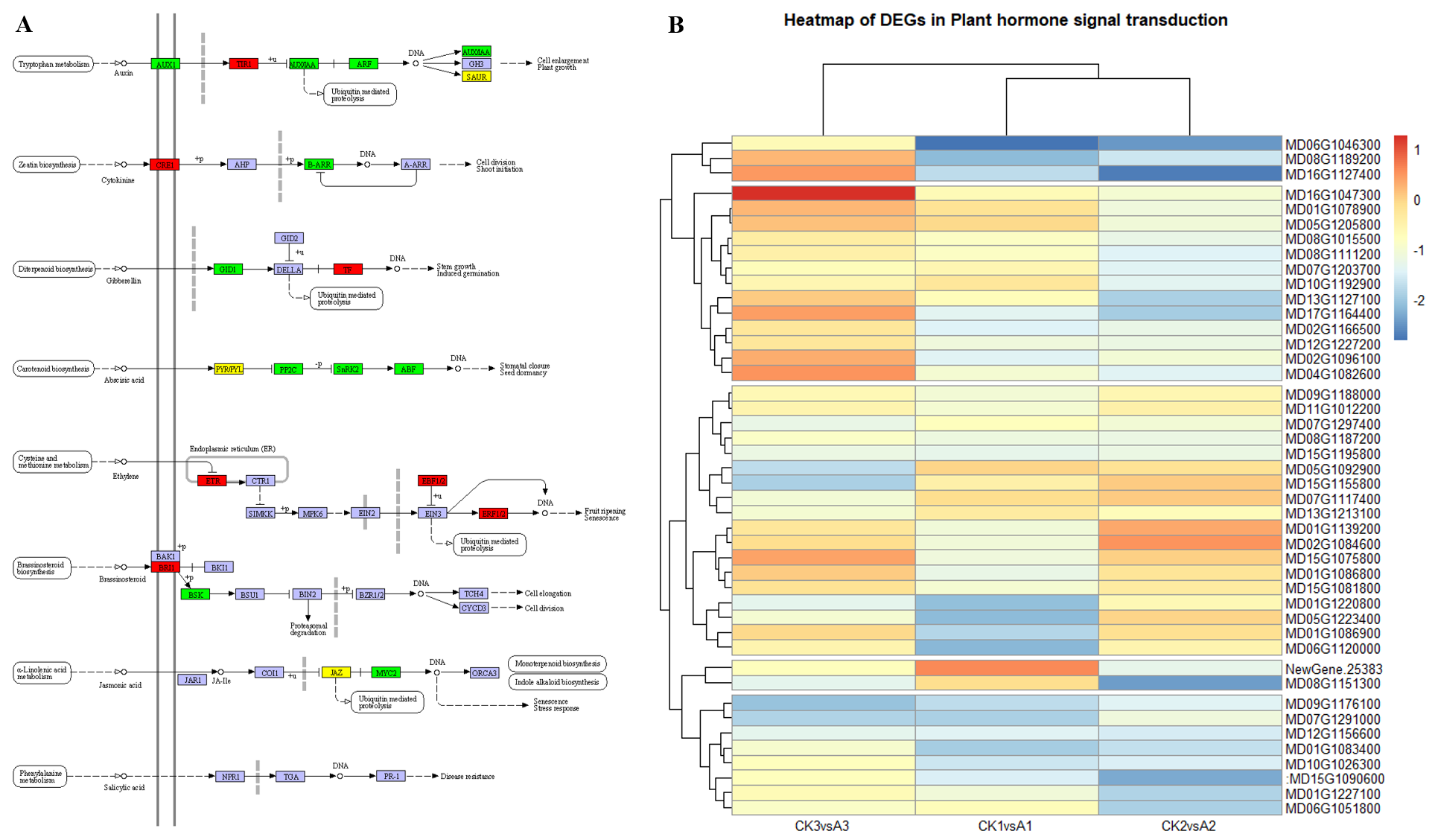

Supplement: S1 Fig — A. Differentially expressed genes enriched in plant hormone signal transduction. Red represents up-regulated genes maped to this node, green represents down regulated genes maped to this node, yellow represents both up-regulated genes and down-regulated genes maped to this node. B. Cluster heat map of differentially expressed genes related to plant hormone signal transduction pathway. The horizontal axis showed the name of different comparison groups, and the vertical axis represent different genes. (TIF) [file pone.0239737.s006.tif]
